# Supplementary material for: Northern bobwhite select for shrubby thickets interspersed in grasslands during fall and winter
Source: PLoS One. 2021 Aug 18;16(8):e0255298. doi: 10.1371/journal.pone.0255298 (PMC8372937; doi:10.1371/journal.pone.0255298)
Supplement: Attachment — Submitted filename: PlosOne_NResource Selection.docx [file pone.0255298.s001.docx]

Below are my line-specific comments.

Lines 40-45: Rather including beta values and credible intervals, I would like to see the probabilities of selection, or magnitudes of effect. Similar to what you present in the results.

Line 80: This is central to northern bobwhite conservation, understanding the spatial arrangement o shrub cover on the landscape. Can you provide any more information about what the interspersion looks like? Or, what those metrics to quantify interspersion are? This measurement of heterogeneity is something you discuss, but don’t formally quantify or evaluate.

Line 82: What kind of forest? Deciduous, oak, pine savannah?

Line 93: Nice to have these percent cover numbers for reference, but if your discussing the importance of heterogeneity, what is the arrangement of these patches? What if all of the forb, litter, and bare ground are spatially clumped together. Will that suffice?

Line 99: Yes! Good definition. But how do you quantify the amount of non-uniformity?

Line 112: You set-up this intensive (traditional) vs. extensive dichotomy but do not really follow through with it. If it is central to this paper, you should continue to relate your results and discussion to the 2 different management styles.

Line 114: You need to define what these categories are.

Line 115: What is in the food plots?

Line 119: Define these things, what structure do they create? Is all brush-hogging done the same and create the same structure?

Line 121: You reiterate the importance of spatial arrangement.

Line 125: Check the definitions outlined by Hall et al. 1997 “The habitat concept and a plea for standard terminology.

Line 129: I like that you clearly provide objectives and hypotheses. For number 5 provide specifics for moderate and high values.

Line 170: Again, please define these management provide more specifics. How was the brush-hogging done? What herbicide was used? What is the difference between haying and clipping?

Line 174: What are some of the row-crops?

Line 243: I would like to see these vegetation types defined. Some kind of a description would be helpful. Same can be said for the management actions. Describe these please.

Lime 274: I like this variable for woody cover, but it still doesn’t quantify general heterogeneity.

Line 293: How did you ensure that “random” locations were actually available for bobwhite use (See Hall paper)? What if a “random” locations was generated (in an area inaccessible to northern bobwhite) that was not available for the birds to use? I know the random locations make sense statistically, but if they are truly random then they might not be “available” to the birds. Please clarify this, or adjust your terminology.

Line 337: How did you model the intensive and extensive management styles? Did you account for these categories at all?

Line 394- Is the range really similar? Doesn’t seem to be. I would delete this sentence.

Line 398- Check the wording, it’s mixed up. Also, how much less? Provide magnitudes of effects.

Line 398- Also, did you do any “stats” on these differences? You could run a t-test to determine if the covariates are significantly different. Otherwise, what is your criteria for determining if used and available are “different”?

Line 415: I appreciate the details included about the linear model and parameters, but how did you construct the individual models? Did you use a stepwise procedure? What was your crietria for including or removing parameters?

Line 447: I like the way you present the effect size of the results.

Table 2: To my point above, how did you decide on these models?

Line 541: But you didn’t evaluate any heterogeneity metrics. How does your research support anything about heterogeneity? Woody Edge Density is the closest you get to quantifying interspersion.

Line 546: During what time of year?

Line 553: Again, be careful about using the term heterogeneity.

Line 578: How was interspersion defined and measured? I thought you did percent cover? You can quantify interspersion, but that isn’t what you did in this study.
