# Supplementary material for: Northern bobwhite select for shrubby thickets interspersed in grasslands during fall and winter
Source: PLoS One. 2021 Aug 18;16(8):e0255298. doi: 10.1371/journal.pone.0255298 (PMC8372937; doi:10.1371/journal.pone.0255298)
Supplement: Attachment — Submitted filename: ResponsetoReviewers.docx [file pone.0255298.s002.docx]

Manuscript ID PONE-D-20-33262

Academic editor and reviewer comments to the authors

*Author responses italicized and in red*

Both subject experts expressed concern over lack of clarity in the statistical methods. In addition, Reviewer 1 suggests that the hypotheses being tested might be better addressed with several tweaks to the overall modeling approach. Please address the statistical critique in your revised draft and response.

In your Methods section, please provide additional location information of the study sites, including geographic coordinates for the data set if available.

*Response: We included the geographic coordinates of each study site (lines 171-175).*

3.) In your Methods section, please provide additional information regarding the permits you obtained for the work. Please ensure you have included the full name of the authority that approved the field site access and, if no permits were required, a brief statement explaining why.

*Response: We include a statement indicating that all animal handling procedures were approved by the University of Missouri Animal Care and Use Committee protocol #8766. No additional permits were required as the Missouri Department of Conservation was a collaborator on this project (lines 241-242).*

4.) We note that you have stated that you will provide repository information for your data at acceptance. Should your manuscript be accepted for publication, we will hold it until you provide the relevant accession numbers or DOIs necessary to access your data. If you wish to make changes to your Data Availability statement, please describe these changes in your cover letter and we will update your Data Availability statement to reflect the information you provide.

*Response: We have submitted data to the U.S. Department of Agriculture Forest Service Research Data Archive. We will provide accession numbers once the data are accepted.*

5.) We note that Figure 1 in your submission contains map images which may be copyrighted. All PLOS content is published under the Creative Commons Attribution License (CC BY 4.0), which means that the manuscript, images, and Supporting Information files will be freely available online, and any third party is permitted to access, download, copy, distribute, and use these materials in any way, even commercially, with proper attribution. For these reasons, we cannot publish previously copyrighted maps or satellite images created using proprietary data, such as Google software (Google Maps, Street View, and Earth). For more information, see our copyright guidelines: <http://journals.plos.org/plosone/s/licenses-and-copyright>.

*Response: No part of this figure is copyrighted.*

Lines 40-45: Rather including beta values and credible intervals, I would like to see the probabilities of selection, or magnitudes of effect. Similar to what you present in the results.

*Response: Thank you for the suggestion, we added the probabilities of selection (lines 41-51).*

Line 80: This is central to northern bobwhite conservation, understanding the spatial arrangement of shrub cover on the landscape. Can you provide any more information about what the interspersion looks like? Or, what those metrics to quantify interspersion are? This measurement of heterogeneity is something you discuss, but don’t formally quantify or evaluate.

*Response: We added definitions of heterogeneity and interspersion for clarity while simultaneously reducing the usage of these terms (lines 93-96, 98-100).*

Line 82: What kind of forest? Deciduous, oak, pine savannah?

*Response: In this context, it could be any of the above which is why we use the general term forests. However later in the study area section we specifically mention deciduous forest (line 184).*

Line 93: Nice to have these percent cover numbers for reference, but if your discussing the importance of heterogeneity, what is the arrangement of these patches? What if all of the forb, litter, and bare ground are spatially clumped together. Will that suffice?

*Response: Later in the paragraph we now define the concept of heterogeneity and the importance of interspersion and proximity to woody cover (lines 93-96).*

Line 99: Yes! Good definition. But how do you quantify the amount of non-uniformity?

*Response: We quantify heterogeneity through the use of percent cover and distance to or from a given cover type. We have indicated this in the manuscript (lines 125-127).*

Line 112: You set-up this intensive (traditional) vs. extensive dichotomy but do not really follow through with it. If it is central to this paper, you should continue to relate your results and discussion to the 2 different management styles.

*Response: Thank you for pointing out this mis-alignment. Our focus was not to compare intensive and extensive management techniques. We described these management approaches in the Introduction to provide context but have now removed it from further discussion (151-152).*

Line 114: You need to define what these categories are.

*Response: We added definitions of categories (lines 138-144).*

Line 115: What is in the food plots?

*Response: The food plots typically contain agricultural crops such as corn, wheat, and soybeans. We added an explanation of food plots to improve clarity (lines 132).*

Line 119: Define these things, what structure do they create? Is all brush-hogging done the same and create the same structure?

*Response: Prescribed fire removes all vegetation and litter, creating bare ground. Grazing reduces the height of vegetation. Brush hogging removes shrubs while leaving litter on the site. Haying and high clipping both reduce the height of grasses and forbs; however haying removes litter from the site while high clipping does not. Herbicides kill unwanted vegetation while leaving litter on site. We included explanations of the structure created by the different management techniques (lines 138-144).*

Line 121: You reiterate the importance of spatial arrangement.

*Response: Thank you for pointing out this redundancy. We have removed the sentence.*

Line 125: Check the definitions outlined by Hall et al. 1997 “The habitat concept and a plea for standard terminology.

*Response: Thank you for your suggestion. We have revised the manuscript to indicate that we measured vegetation types and made every effort to take care on use of the terms habitat type, cover, and cover types (line 147).*

Line 129: I like that you clearly provide objectives and hypotheses. For number 5 provide specifics for moderate and high values.

*Response: We added specific values (lines 165-166).*

Line 170: Again, please define these management provide more specifics. How was the brush-hogging done? What herbicide was used? What is the difference between haying and clipping?

*Response: We included more precise definitions of each management technique (lines 194-200).*

Line 174: What are some of the row-crops?

*Response: We edited to include examples of row crops: corn, soybeans, wheat (line 199).*

Line 243: I would like to see these vegetation types defined. Some kind of a description would be helpful. Same can be said for the management actions. Describe these please.

*Response: We included definitions of vegetation types and management techniques (lines 267-277).*

Lime 274: I like this variable for woody cover, but it still doesn’t quantify general heterogeneity.

*Response: We previously clarified how our use of percent cover in combination with distance to woody cover gets at heterogeneity (lines 308-309).*

Line 293: How did you ensure that “random” locations were actually available for bobwhite use (See Hall paper)? What if a “random” locations was generated (in an area inaccessible to northern bobwhite) that was not available for the birds to use? I know the random locations make sense statistically, but if they are truly random then they might not be “available” to the birds. Please clarify this, or adjust your terminology.

*Response: Thank you for the suggestion. Locations were assumed available to the birds because they were within the distance travelled between resightings of bobwhite within our study. We added a few words to help clarify (lines 331-334).*

Line 337: How did you model the intensive and extensive management styles? Did you account for these categories at all?

*Response: This is a great point. The purpose of this paper was not to evaluate the differences in intensive and extensive management styles. We have included a definition in the introduction for context and then removed further mention and focused instead on the variables that we modeled (lines 372).*

Line 394- Is the range really similar? Doesn’t seem to be. I would delete this sentence.

*Response: We removed the sentence (line 437).*

Line 398- Check the wording, it’s mixed up. Also, how much less? Provide magnitudes of effects.

*Response: We corrected the wording (lines 440-442).*

Line 398- Also, did you do any “stats” on these differences? You could run a t-test to determine if the covariates are significantly different. Otherwise, what is your criteria for determining if used and available are “different”?

*Response: This is just a simple descriptive comparison of means and we reworded it to indicate so. Our model provides a more rigorous analysis of selection (line 437).*

Line 415: I appreciate the details included about the linear model and parameters, but how did you construct the individual models? Did you use a stepwise procedure? What was your crietria for including or removing parameters?

*Response: Variables of interest were chosen based on our hypotheses and recent results from a complementary study on bobwhite survival at these sites. We included an explanation of this (line 378-380).*

Line 447: I like the way you present the effect size of the results.

*Response: Thank you.*

Table 2: To my point above, how did you decide on these models?

*Response: Variables of interest were chosen based on recent results from a parallel study on bobwhite survival at these sites. We have included an explanation of this (lines 378-380).*

Line 541: But you didn’t evaluate any heterogeneity metrics. How does your research support anything about heterogeneity? Woody Edge Density is the closest you get to quantifying interspersion.

*Response: We included a definition of heterogeneity and how it applies to this work. We also reduced the focus on the term heterogeneity. Instead, we refer to our terms exactly as they were calculated to improve clarity (lines 585-586).*

Line 546: During what time of year?

*Response: The cited research examined resource use throughout the brood rearing and nesting season. We included the time of year in the revised manuscript (line 590).*

Line 553: Again, be careful about using the term heterogeneity.

*Response: Thank you for the comment. We have made every effort to take care on use of heterogeneity (described further above; lines 597-598).*

Line 578: How was interspersion defined and measured? I thought you did percent cover? You can quantify interspersion, but that isn’t what you did in this study.

*Response: The concept of interspersion is addressed by several variables so we reduced the mention of the term interspersion and instead focused on the exact metrics that we calculated (distance to shrubs, distance to trees, woody edge density; line 622).*

It seems as though you had specific hypotheses that bobwhite might be selecting habitat differently in fall and winter (thus including fall and winter as a fixed effect) – this seems like a valid approach and you describe many reasons for this in the introduction. However, by including fall/winter as a binary fixed effect and not modelling resource selection separately, aren’t you constraining your results such that one model with all the same variables is produced for fall and winter (i.e., there is no possibility that a different set of variables could be important in fall and winter – e.g., trees might be important in fall but not at all in winter)? Why not just conduct separate resource selection analyses for data from fall and winter, instead of combing into one analysis?

*Response: You are correct that our approach considers the same variables for fall and winter within a model, however, separate effects are estimated for fall and winter for each covariate within the model. So the effect can vary between seasons, and in fact we observed this for some covariates. Splitting the data and developing separate models is an alternative, but given that there were more similarities than differences between seasons we think there is more to be gained by keeping the data together in one model.*

It is unclear how you chose the 6 models displayed in Table 2 – the process of identifying models m1-m6 is not described in the methods. For example it does not appear that you examined a model with all the variables (i.e., a global model) – was this on purpose?

*Response: We were not able to fit a global model due to limitations in the number of estimable parameters per model. We have included an explanation in the revisions (lines 402-403).*

It appears as though you went through great lengths to capture and select bobwhite from intensively managed and extensively managed landscapes. But then birds from each of these groups are essentially lumped together for all analyses. Is this because the “discrete choice” modelling takes that information into account and can deal with issues such as different habitat availability etc? I would assume that if bobwhites only used the native habitats in the intensively managed landscapes and the choices they had were all non-native habitats, you would see a “strong” pattern of selection. Whereas if bobwhites used native habitats in the extensively managed landscapes and their only choices were other native habitats you might observe no selection (your used points and random points would have grass at all of them)?And then by combining them you’d end up with a result somewhere in the middle - Is this an issues in the analyses or does the discrete choice modelling tackle that issue?

*Response: Our analysis compares covariates within a choice set of 1 used location and 3 available locations. These choice sets can vary over time and between coveys so availability is always defined for a given individual at a point in time. The differences in extensive versus intensive study sites provides the management context for our study but most importantly it creates a range of covariate values across individuals for our study which allows evaluation of resource selection more broadly, across intensive and extensively managed sites.*

Line 65-66: I’m not sure trade-off is exactly the correct term to use here. There may not be a trade-off and habitat selection may be exactly the same in all seasons (or you might be able to manage the landscape such that no habitat is lost at the expense of creating other habitat). Do you mean “to refine management decisions” based on information for other seasons?

*Response: Yes, you are correct. We edited the sentence following your suggestion to improve clarity (line 70).*

Line 71: Is there another term besides “rank” that you could use? That may not be a term that is familiar to everyone. Would “overly tall and dense” be appropriate?

*Response: Yes, you are correct that the term rank may not be familiar to everyone. Your suggestion is appropriate and we edited the sentence to reflect your suggestion (line 76).*

Paragraph starting on line 168: This paragraph is filled with very “jargony” habitat management terms making it difficult to understand (e.g., what is brush hogging, high clipping, what are food plots?). The terms extensively managed and intensively managed also do not seem like particularly common terms – please explain. It appears as though the native grassland landscapes are managed to try to keep them native, whereas the intensively managed landscapes are composed of non-native habitat. There also seems to be a bit of a contradiction in the intensively managed landscapes – you list all non-native habitat types in lines 174-175, but then go on to say that some of these fields were converted back to native prairie, so why not just list native prairie as a cover type?

*Response: We revised “brush hogging” to “mechanical brush removal” throughout the paper. We added an explanation that high clipping is mowing grasses to a height of ≥30 cm. We included a description of food plots. Extensive and intensive management refer to differences in management techniques used to achieve suitable bobwhite habitat. These terms are further explained in the 6^th^ paragraph of the Introduction, and we have included clarifying terminology there. We noted that some fields were converted back to native prairie on Shawnee and Talbot because these are different than the remnant, virgin prairies which occur on Stoney, Shelton, and Wah’Kon-Tah (lines 191-204).*

Paragraph starting on line 240: Some similar issues as my previous comment. For readers not from Missouri these terms are somewhat confusing (what is the difference between native prairie, native grass planting and restored prairie). This will help to understand why you grouped these together.

*Response: Thank you for the suggestions. We included definitions to increase clarity (lines 266-277).*

Line 300-301: I am a bit unclear as to why some of these variables (e.g., agricultural crops, woody cover) were measured via a Daubenmire frame and not just extracted from your landuse/landcover maps? Wouldn’t the landcover mapping be more appropriate?

*Response: We included these different measures because we wanted to evaluate relationships of composition and structure at several spatial scales to get at the ideas of landscape versus local heterogeneity.*

Line 332: the "NG PG" term should be explained in this paragraph as well. I assume this is an interactive effect?

*Response: That is correct. The term NG PG is an interaction between NG (percent native grass within 50 m) and PG (percent of land managed with either prescribed burning or grazing within 50 m). We included the definition for NG PG (lines 374-375).*

Paragraph starting on line 517: Could the bobwhite be attracted to trees and shrubs in winter to provide some degree of cover from the elements, but are at higher risk from predators?

*Response: Yes and that sets up the possibility of an ecological trap (lines 572-575).*

Table 1: Given that there is a difference in selection in fall and winter, this table would be more useful if it provided the means and ranges for fall and winter separated rather than lumped together.

*Response: We included the means and ranges for fall and winter as suggested (line 445).*

Table 2:
- Further explanation is needed in the methods about what the <0.85 and >0.85 posterior distribution indicates. Why is the 0.85 value chosen?

*Response: In the Bayesian framework, the proportion of the posterior distribution that is positive or negative represents the probability of a positive or negative effect, respectively. So in addition to the mean effect and 85% CRI, we also report the proportion of posteriors that were positive or negative when >0.85 of the distribution was above or below zero, as another measure of support. We chose 0.85 as a broader threshold to the traditional 0.95 threshold because we think it more clearly illustrates the likelihood of an effect explaining variation in a response, particularly given complex ecology. With full posteriors from a Bayesian analysis, we encourage other practitioners to report lower thresholds as well. While we used a 0.85 threshold for CRIs and to summarize effects in Table 2, we also present the proportion of the posterior distribution that was positive or negative and plots of the entire posterior distribution so readers have the flexibility to interpret support for relationships using multiple criteria (lines 419-426).*

- Make the figure heading clearer by adding the "fall/winter" after the sentence concerning the directions of effect. It’s not clear when you refer to “fall/winter” in the sentence previous that is it referring to how the table is structured.

*Response: We made the suggested change (lines 476).*

First, they do a good job of detailing the linear model equation, and what each parameter represents. But, how did they decide on the variables within models? They state that they used a VIF threshold for combinations of variables but not how they determine combinations. Was every variable combination checked? What process was used for creating the combinations of parameters?

*Response: Parameters were chosen based on what was important to bobwhite survival in a parallel study. We explained this and cited the MS thesis as the survival paper is not yet published (line 378-380).*

Second, the researchers don’t formally quantify heterogeneity. They measure percent cover of different cover types, and they include disturbance regimes and the land-use history, but they don’t quantify the arrangement of the cover types within their sample areas. There are ways to quantify heterogeneity. Interspersion juxtaposition, perimeter to area ratios, coefficient of variation, etc.. I would avoid using terminology like “heterogeneous” unless it is explicitly defined for the context of your research. As it is, your discussion and interpretation of the significance of heterogeneity is inappropriate and does not correspond to your analyses. You can draw inference from the amount of cover available and used.

*Response: Thank you for the suggestions. We agree and have made every effort for greatest transparency of the terms we explicitly evaluated in our analysis and manuscript. We defined heterogeneity in the Introduction and reduced use of the term thereafter to improve clarity on the scope of our work (lines 585-586).*
